# Conservation Genomic Analysis of the Croatian Indigenous Black Slavonian and Turopolje Pig Breeds

**DOI:** 10.3389/fgene.2020.00261

**Published:** 2020-03-31

**Authors:** Boris Lukić, Maja Ferenčaković, Dragica Šalamon, Mato Čačić, Vesna Orehovački, Laura Iacolina, Ino Curik, Vlatka Cubric-Curik

**Affiliations:** ^1^Department for Animal Production and Biotechnology, Faculty of Agrobiotechnical Sciences Osijek, J.J. Strossmayer University of Osijek, Osijek, Croatia; ^2^Department of Animal Science, Faculty of Agriculture, University of Zagreb, Zagreb, Croatia; ^3^Ministry of Agriculture, Zagreb, Croatia; ^4^Department of Chemistry and Bioscience, Aalborg University, Aalborg, Denmark; ^5^Department for Apiculture, Wildlife Management and Special Zoology, Faculty of Agriculture, University of Zagreb, Zagreb, Croatia

**Keywords:** genomics, diversity, inbreeding, local breeds, population structure

## Abstract

The majority of the nearly 400 existing local pig breeds are adapted to specific environments and human needs. The demand for large production quantities and the industrialized pig production have caused a rapid decline of many local pig breeds in recent decades. Black Slavonian pig and Turopolje pig, the latter highly threatened, are the two Croatian local indigenous breeds typically grown in extensive or semi-intensive systems. In order to guide a long-term breeding program to prevent the disappearance of these breeds, we analyzed their genetic diversity, inbreeding level and relationship with other local breeds across the world, as well as modern breeds and several wild populations, using high throughput genomic data obtained using the Illumina Infinium PorcineSNP60 v2 BeadChip. Multidimensional scaling analysis positioned Black Slavonian pigs close to the UK/North American breeds, while the Turopolje pig clustered within the Mediterranean breeds. Turopolje pig showed a very high inbreeding level (F_ROH__>__4__Mb_ = 0.400 and F_ROH__>__8__Mb_ = 0.332) that considerably exceeded the level of full-sib mating, while Black Slavonian pig showed much lower inbreeding (F_ROH__>__4__Mb_ = 0.098 and F_ROH__>__8__Mb_ = 0.074), indicating a planned mating strategy. In Croatian local breeds we identified several genome regions showing adaptive selection signals that were not present in commercial breeds. The results obtained in this study reflect the current genetic status and breeding management of the two Croatian indigenous local breeds. Given the small populations of both breeds, a controlled management activity has been implemented in Black Slavonian pigs since their commercial value has been recognized. In contrast, the extremely high inbreeding level observed in Turopolje pig argues for an urgent conservation plan with a long-term, diversity-oriented breeding program.

## Introduction

For thousands of years, pigs have been indispensable to humans as they represent an important part of our everyday diet. Pigs were domesticated about 8,500–10,500 years ago (Peters et al., 2005; Zeder, 2017) and have changed over time from their wild ancestors, especially in the last few hundred years due to the force of artificial selection. Nearly 400 local breeds have been obtained. Pig populations continue to change genetically because of continuous gene flow between wild and domestic pigs (Iacolina et al., 2018; Frantz et al., 2019). Most local breeds are adapted to specific environments, production systems, geographical regions or human demands. However, in the last few decades, several breeds such as Large White, Duroc, Landrace, Hampshire and Pietrain (FAO, 2007) and their hybrids have spread internationally and replaced most local breeds, mainly because they are economically more efficient. This poses a problem: the conservation of local breeds is crucial for the future of animal production as they can be important sources of genetic variability (Bruford et al., 2015) and are better adapted for production in sustainable environments (Ollivier et al., 2005).

In Croatia, the two indigenous breeds, Black Slavonian (CROBS) and Turopolje (CROTS) pigs, have specific phenotypic characteristics that make them well adapted to the extensive and semi-intensive systems common in the country. The Black Slavonian breed is also known as Fajferica and was bred by the earl Karl Pfeiffer in the second half of the 19th Century in Slavonia, a “corn belt” region in Eastern Croatia. By crossing the local Mangalitza gilts with Berkshire boars, Pfeiffer created a new breed with more desirable economically important traits (feed conversion ratio, daily gain, carcass traits) than the local dominant, primitive breeds such as Mangalitza, Šiška and Bagun. Some years later, the breed was further improved by crossing the best gilts with imported USA Poland China boars. In the 1930’s and 1940’s, the breed was crossed again with Berkshire, and later with Large Black on several farms (Hrasnica et al., 1958). The breed was economically successful, well known for its fat and meat production and one of the most abundant (>300,000 individuals) in Yugoslavia in the 1950’s (Hrasnica et al., 1958). Since then, however, the Black Slavonian pig is slowly being replaced by modern breeds such as Landrace, Large White, and Pietrain. The Black Slavonian population declined drastically at the beginning of the 1990’s, during and after the war in Croatia. The first conservation program with pedigree recording started in 1996 in the founding population, consisting of the only remaining 46 sows and six boars (Uremović, 2004).

The Turopolje pig breed, for its part, is named after a small region near Zagreb (Turopolje), and has a controversial history. A publication from 1911 (Ulmansky, 1911) asserted that CROTS is a cross between local pigs and Šiška, a primitive regional breed currently extinct. A study from 1935 (Ritzoffy, 1935) claimed that Turopolje pig was most probably derived from the Slovenian Krškopolje pig at the beginning of the 19th century, while more recent work (Porter, 2002) has claimed that this breed originated from Šiška, Krškopolje and Berkshire pigs. During the second half of the 20th century, when modern breeds were intensively imported, the Turopolje breed declined severely, like many local European pig breeds. Its survival was also seriously threatened during the war in the 1990’s (Druml et al., 2012).

A sustainable breeding program might prevent further erosion of the genetic adaptive capacity of both Croatian indigenous breeds and lead to more stable populations. The Black Slavonian breed is better positioned than the Turopolje breed as their carcass traits better suit today’s market demands, while the Turopolje breed is a typical lard type of pig that is no longer profitable for farmers, although a recent study showed the breed has potential for some commercially relevant traits (Muñoz et al., 2018). A prerequisite for building a long-term sustainable local breeding program is detailed molecular and genomic characterization. Studies have already explored the population genetic background for the Black Slavonian breed using pedigree information (Lukić et al., 2015), microsatellite markers for both Black Slavonian and Turopolje pigs (Druml et al., 2012; Šprem et al., 2014) and selected single-nucleotide polymorphisms (SNPs) associated with morphological traits (Muñoz et al., 2018). However, a wider and more systematic approach is required to obtain more thorough understanding of their breed genetics.

Recent advances in genotyping technologies, such as SNP chips, provide affordable access to genotypic information for all major domestic animal species, enabling the estimation of the genetic diversity, population structure, genetic admixture, inbreeding level, and effective population size (Kukučková et al., 2017). In addition, SNP data can be merged and compared with the results of other studies, which is impossible with microsatellite marker analyses. Analysis of the frequencies of a large number of SNP alleles provides deep insight into genetic variability and genetic structure. For instance, the genomic inbreeding coefficient obtained from SNP analyses is more reliable than pedigree estimates (Ferenčaković et al., 2013; extensively described by Keller et al., 2011). Genetic admixture, a phenomenon that occurs when genetically divergent populations begin to interbreed (Balding et al., 2007), is conventionally identified by multivariate genetic cluster algorithms (Jombart et al., 2010).

Taking advantage of high-throughput genomic analyses, we explored the genetic structure of Black Slavonian and Turopolje pig breeds and their relationships with local and modern breeds worldwide. We also estimated the inbreeding level based on runs of homozygosity (ROH) as well as admixture level, particularly important for the Turopolje pig, which is classified as a highly endangered breed (Croatian Agricultural Agency, 2017). For each indigenous Croatian breed, we identified a set of SNPs that differentiate it from the most widespread modern commercial breeds. These results may inform future conservation management of Black Slavonian and Turopolje pigs.

## Materials and Methods

### Data Collection, Quality Control, and Multidimensional Scaling

The animals in this study were selected in collaboration with the Croatian Agricultural Agency, which is the national body that manages breeding programs, and the National Gene Bank within the Ministry of Agriculture of Croatia. All procedures with animals were performed in accordance with national and European ethical protocols and directives. Animals were raised by registered breeders at more than five locations, with available information about their origin. In the case of Black Slavonian pigs, sampling of close relatives (parent-offspring, full sibs or half sibs) was avoided. In the case of Turopolje pigs, animals were sampled at random because the population was extremely small (124 sows and 17 boars; Croatian Agricultural Agency, 2017) and contained many higher-order relatives. In this case, avoiding sampling of close relatives would lead to biased results. More detailed information describing samples in this study is provided in Supplementary Table S1.

A total of 16 Black Slavonian pigs (six boars and 10 sows) and 16 Turopolje pigs (four boars and 12 sows) were genotyped using Illumina PorcineSNP60 v2 Genotyping BeadChip with 64,232 SNPs (Ramos et al., 2009). DNA was isolated from hair follicles using a commercial kit (DNeasy Blood and Tissue Kits, Qiagene, Germany). Using the obtained genotypes, we analyzed only autosomal SNPs whose chromosomal position was assigned. SNPs where more than 10% of genotypes were missing and SNPs with Illumina GenCall score ≤ 0.7 or Illumina GenTrain score ≤ 0.4 (Ferenčaković et al., 2013) were excluded from the analysis. Pigs for which > 5% of the genotype was missing were also excluded from further analysis. SNP positions were based on the pig genome assembly Sscrofa 10.2 (EnsEMBL db version 83). In order to compare our data with worldwide data sets, additional data (Ai et al., 2013; Burgos-Paz et al., 2013; Goedbloed et al., 2013) were downloaded from the publicly available Dryad Digital Repository (Yang et al., 2017).

**FIGURE 1:**
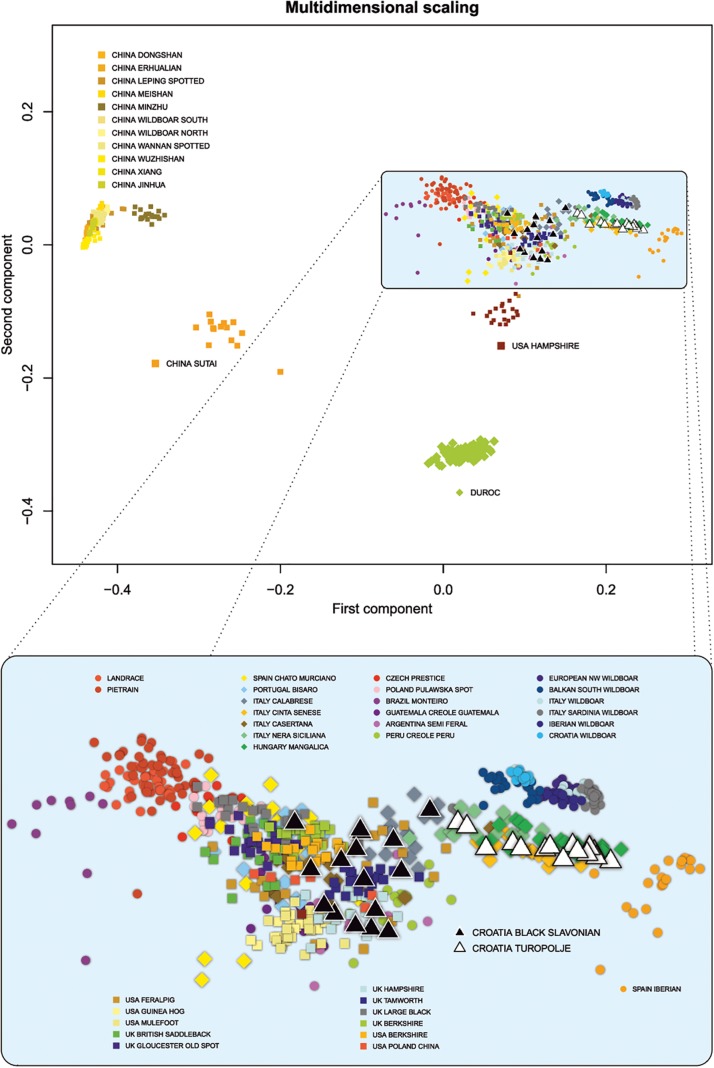
Population structure of the worldwide pig breeds and wild boar populations analyzed by multidimensional scaling (MDS). All breeds/populations are presented in the upper part of the illustration while selection of 32 pig and wild boar populations is presented in the lower part of the illustration.

We used several criteria to select breeds from public data. First, we selected breeds known to share a history with Croatian local breeds (e.g., founder breeds) or to inhabit areas close to those of Croatian breeds. Second, breeds with similar phenotypic traits such as coat color or exterior traits were selected, since such traits were among the main selection criteria during early stages of animal breeding. Genetic similarity of local breeds with wild boar is expected to be high, so we included several wild European populations. Chinese breeds were also included because of their known introgression into the international gene pool, and particularly into the commercially important breeds Landrace, Pietrain, and Duroc. This data set was then merged with our samples to produce a consensus data set containing 931 animals from 48 breeds (of which nine were wild boar populations) and 45,000 SNPs. SNP genotypes were used to calculate shared genetic coancestry between all possible pairs of individuals of all breeds in the analysis in terms of pairwise proportions of identical-by-state alleles using R software version 3.6.1 (R Core Team, 2019). The obtained matrix was transformed to a distant matrix, on which classical multidimensional scaling and principal component analysis were performed. This analysis showed that Chinese breeds, USA Feral Pig, Argentina Semi Feral Pig, Brazil Monteiro Pig, Guatemala Creole Pig, Peru Creole Pig, USA Guinea hog, USA Mulefoot, and Duroc form distant clusters (Figure 1). To provide better resolution and more precise characterization of the Black Slavonian and Turopolje pigs, breeds present in distant clusters were removed from subsequent analyses.

The final data set consisted of 556 animals sampled from 30 breeds, including six wild boar populations. Landrace and Pietrain breeds were represented by two different populations to provide additional controls. The following breeds were used in the analyses: Black Slavonian – CROBS *(n* = 16), Croatian Wild Boar – CROWB (16), Czech Prestice – TRPR (15), German Angler Sattleschwein – DEAS (*n* = 10), Hungarian Mangalitza – HUMA (*n* = 20), Iberian Wild Boar – IBWB (*n* = 17), Italian Calabrese – ITCA (*n* = 15), Italian Casertana – ITCT (*n* = 14), Italian Cinta Senese – ITCS (*n* = 13), Italian Nera Siciliana – ITNS (*n* = 15), Italian Sardinian Wild Boar – ITWB2 (*n* = 20), Italian Wild Boar – ITWB1 (*n* = 19), Landrace population 1 – LDR1 (*n* = 20), Landrace population 2 – LDR2 (*n* = 15), NW European Wild Boar – NEWB (*n* = 20), Pietrain population 1 – PIT1 (*n* = 20), Pietrain population 2 – PIT2 (*n* = 20), Polish Pulawska Spot – PLPS (*n* = 15), Portuguese Bisaro – PTBI (*n* = 14), South Balkan Wild Boar – SBWB (*n* = 20), Spanish Chato Murciano – ESCM (*n* = 20), Spanish Iberian – ESIB (*n* = 20), Turopolje – CROTS (*n* = 16), UK Berkshire – UKBK (*n* = 20), UK British Saddleback – UKBS (*n* = 20), UK Gloucester Old Spot – UKGO (*n* = 20), UK Hampshire – UKHS (*n* = 20), UK Large Black – UKLB (*n* = 20), UK Tamworth – UKTA (*n* = 20), USA Berkshire – USBK (*n* = 20), USA Hampshire – USHS (*n* = 20), and USA Poland China – USPC (*n* = 6). Sample sizes for all breeds were similar with the exception of the Poland China breed.

### Genetic Admixture

The population structure and admixture analyses were performed on the final data set using a Bayesian approach implemented in STRUCTURE software 2.3.4 (Pritchard et al., 2000) without prior information about the population. We had to reduce the number of SNP genotypes in the dataset to 15,000 to enable the complex computations, which otherwise would not have been possible. In order to estimate global ancestry, we used a model with assumed admixture and correlated allele frequencies, as this provides greater power to reveal populations that are closely related (Porras-Hurtado et al., 2013). We performed analyses for the assumed K number of populations from 1 to 34, with 20 independent runs and a burn-in period of 10,000 followed by 100,000 Markov chain Monte Carlo repetitions. The calculations related to STRUCTURE software were performed on the Isabella computer cluster at the University Computing Centre (SRCE) of the University of Zagreb. The choice of the most likely number of clusters (K) was determined according to recommendations in previous work (Pritchard et al., 2000), as well as according to visual representations showing the rate of change in ln Pr(G| K) between successive *K*-values (Evanno et al., 2005). Clumpak software (Kopelman et al., 2015) was used to estimate the maximum probability from *K* = 1 until *K* = 30 and average the individual results among the 20 runs for each K (Jakobsson and Rosenberg, 2007) and over different *K*-values. The obtained results were visualized using the Pophelper 2.2.7 package for R (Francis, 2017).

### ROH and Genomic Inbreeding

The ROH-based genomic inbreeding coefficient (F_ROH_) was calculated as described (McQuillan et al., 2008; Curik et al., 2014), where F_ROH_ = genome length in ROH/autosomal genome length covered by the SNP chip (here 2,444.5 Mb spread over 18 chromosomes). Based on the SNP density of the Illumina PorcineSNP60 v2 Genotyping BeadChip and the 45,000 SNPs remaining after quality control, ROH were called if 15 or more consecutive homozygous SNPs were present at a density of at least one SNP every 0.1 Mb, with gaps of no more than 1 Mb between them. ROH segments were detected using cgaTOH software (Zhang et al., 2013). To identify ROH segments, we allowed one, two and four missing calls per window, respectively, for ROH > 4 Mb, ROH > 8 Mb, and ROH > 16 Mb (Ferenčaković et al., 2013). This approach identifies ROHs according to the length size class. By merging the information related to each class, we were able to calculate genomic inbreeding coefficients (F_ROH__>__4__Mb_ and F_ROH__>__8__Mb_). Additionally, we calculated F_ROH__4__to__8__Mb_ as the difference between F_ROH__>__4__Mb_ and F_ROH__>__8__Mb_. In this way, we were able to distinguish F_ROH__>__4__Mb_ from “remote” inbreeding (F_ROH__4__to__8__Mb_) arising from ancestors approximately –13 generations remote as well as from “recent” inbreeding (F_ROH__>__8__Mb_) arising within the last seven generations (Kukučková et al., 2017).

### Population Structure and Differentiation of Populations

Global genetic differentiation between the two Croatian local breeds, as well as between the Croatian breeds and other world populations, was assessed in terms of the genome wide fixation index, F_ST_, for each SNP pair (Weir and Cockerham, 1984). This index was calculated in Plink (Purcell et al., 2007) and GenePop Version 4.7.0 (Rousset, 2008). We also illustrated genetic divergence among breeds/populations by the neighbor-joining tree (NJ) based on Reynold’s distances matrix (Reynolds et al., 1983). Reynolds genetic distances were calculated using Arlequin 3.5 (Excoffier and Lischer, 2010) software, which were used to construct a neighbor-joining tree in R package phytools (Revell, 2012).

### Identification of Adaptive Signatures of Selection

In order to identify SNP alleles with high F_ST_ values specific to Croatian local breeds, we created two additional datasets, one composed of Black Slavonian and modern commercial breeds (Landrace and Pietrain), and another with Turopolje pigs and the same modern commercial breeds. Based on the two analyses, we selected 30 genome-wide SNPs with the highest F_ST_ values for Black Slavonian and Turopolje pigs (Supplementary Figures S1, S2 and Supplementary Tables S2, S3). The Ensembl Genome Browser^1^ was used to identify candidate genes in 0.1 Mb wide genomic regions with high F_ST_ values. In order to explore and confirm the signals of the adaptive positive selection, we performed additional analyses: (a) identification of extremely frequent SNPs in ROHs (eROHi) approach (Curik et al., 2014); (b) extended haplotype homozygosity (EHH) approach (Sabeti et al., 2002) modified as within population Integrated Haplotype Score (iHS) approach (Voight et al., 2006) and (c) across populations Integrated Haplotype Score (Rsb) approach, based on the ratio of site-specific EHH (EHHS) between populations (Tang et al., 2007). Similar approach of combining F_ST_ and extremely frequent ROHs was applied by Purfield et al. (2017). Both, iHS and Rsb statistics were calculated and tested in rehh R package (Gautier and Vitalis, 2012) while required phasing was estimated with Shapeit software (Delaneau et al., 2008). The conservative significance threshold of *P* = 0.0001 (equivalent to 10,000 independent tests), defined with −log_10_ (*P*-value) = 4.0, was used in iHS and Rbs tests to account for multiple testing. The eROHi approach has been applied in CROBS, CROTS and commercial pig breeds as our first interest was to identify selection signals that are specific for Croatian local breeds, extreme ROH islands present in CROBS or CROTS but not appearing in commercial breeds. Significant autozygosity islands, SNPs with extreme ROH frequency, were identified as outliers (99%) according to the BOXPLOT distribution as applied in Mészáros et al. (2015). Identified specific regions were then checked for the candidate genes under selection using the free Golden Helix GenomeBrowse^®^ and pig genome assembly Sscrofa 10.2 (EnsEMBL db version 83).

## Results

### Multidimensional Scaling Analysis

In order to analyze the genetic relationship between the Croatian indigenous pig breeds and other worldwide pig breeds or wild boar populations, the MDS approach was used to calculate the shared genetic coancestry among all individuals and breeds/populations (Figure 1). Based on the first and second principal components, four main breed clusters were resolved: two Chinese local breed clusters, a Duroc cluster, and a European and North American cluster containing the two indigenous Croatian breeds. The first component clearly separated the Chinese breeds from the European and North American, whereas the second component split one Chinese breed (Sutai), Duroc and Hampshire from the main Chinese and European/North American cluster. A closer look at the European and North American clusters (Figure 1, lower part) showed that the commercial breeds Landrace and Pietrain were separated from the breeds in the middle group, which was dominated by the UK and North American local breeds. The wild populations also formed a small independent group, close to the larger group dominated by the Italian local breeds. Croatian indigenous breeds grouped close to the old UK breeds and Italian breeds. As expected, Black Slavonian pigs lay close to its UK and USA breeds of origin: USA Poland China, Berkshire and Large Black. The Turopolje pig breed, in contrast, grouped together with the Italian breeds and Mangalitza, in an intermediate position between the UK local breeds and the wild populations.

### Admixture Analysis

**FIGURE 2:**
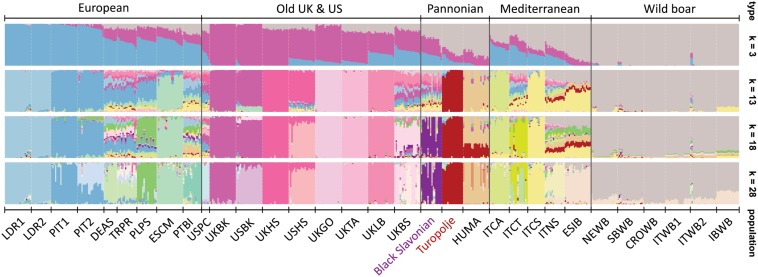
Population structure and admixture for selected 32 pig and wild boar populations illustrated by STRUCTURE bar plots representing models with different clusters (*K* = 3, *K* = 13, *K* = 18, and *K* = 28). The breeds on the plot are: **Black Slavonian**, Croatia Wild Boar – CROWB, Czech Prestice – TRPR, Germany Angler Sattleschwein – DEAS, Hungarian Mangalitza – HUMA, Iberian Wild Boar – IBWB, Italian Calabrese – ITCA, Italian Casertana –ITCT, Italian Cinta Senese –ITCS, Italian Nera Siciliana –ITNS, Italian Sardinian Wild Boar – ITWB2, Italian Wild Boar – ITWB1, Landrace population 1 – LDR1, Landrace population 2 – LDR2, NW European Wild Boar –NEWB, Pietrain population 1 – PIT1, Pietrain population 2 – PIT2, Poland Pulawska Spot – PLPS, Portugal Bisaro –PTBI, South Balkan Wild Boar – SBWB, Spain Chato Murciano –ESCM, Spanish Iberian –ESIB, **Turopolje**, UK Berkshire –UKBK, UK British Saddleback –UKBS, UK Gloucester Old Spot –UKGO, UK Hampshire –UKHS, UK Large Black –UKLB, UK Tamworth – UKTA, USA Berkshire – USBK, USA Hampshire – USHS, and USA Poland China – USPC.

The genetic structure of 32 breeds/populations obtained by the STRUCTURE analysis is presented in Figure 2, while more detailed explanations about this analysis are provided in Supplementary Figure S4. We have presented only results that are relevant for the understanding of the Black Slavonian and Turopolje pig clustering. Thus, the first initial split of *K* = 3 identified a cluster (gray color) belonging to wild populations present also in the Mediterranean and the Pannonian breeds, a cluster (blue) for the European and commercial breeds influencing the Mediterranean more than the Pannonian breeds, and a cluster (pink) for the old UK and US breeds influencing the European and Pannonian breeds more than the Mediterranean ones. At *K* = 13, the Turopolje pig constituted a unique cluster whereas the Black Slavonian breed was identified as a single cluster only from *K* = 18, while it showed high genetic admixture with modern breeds. Despite their geographical proximity, we did not observe any admixture traces between Black Slavonian and Turopolje pigs. In the most likely model of *K* = 28, most of the 24 breeds appeared as individual clusters, except for German Angler Sattleschwein (DEAS) and Czech Prestice (TRPR), which overlapped. All six wild boar populations appeared as a separate group across all K values. However, at *K* = 28, a small amount of the Spanish Iberian (ESIB) component was present in all wild boar populations, particularly in the Iberian Wild Boar (IBWB). At the low level of differentiation (*K* = 3), the largest amount of the wild boar cluster was present in the Mediterranean and Pannonian breeds. One Pietrain population (PIT2), UK Berkshire (UKBS), Hungarian Mangalitza (HUMA) and ITCT (Italian Casertana) showed slight sub-structuring, while the USA Berkshire (USBK) and USA Hampshire (USHS) breeds appeared as more separated in comparison to the other breeds from UK and USA (Figure 2).

### ROH-Based Analysis of Genomic Inbreeding

**FIGURE 3:**
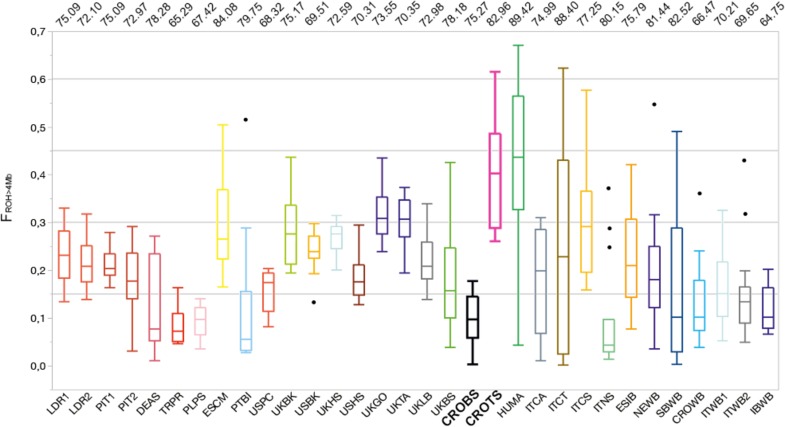
The distribution of the ROH inbreeding coefficients (F_ROH__>__4__Mb_) for selected 32 pig and wild boar populations. Numbers on the top of the illustration present the contribution (%) of the “close” inbreeding (F_ROH__>__8__Mb_) caused by ancestors within seven generations relative to the total inbreeding level (F_ROH__>__4__Mb_). The breeds on the plot are: **Black Slavonian – CROBS**, Croatian Wild Boar – CROWB, Czech Prestice – TRPR, German Angler Sattleschwein – DEAS, Hungarian Mangalitza – HUMA, Iberian Wild Boar – IBWB, Italian Calabrese – ITCA, Italian Casertana – ITCT, Italian Cinta Senese – ITCS, Italian Nera Siciliana – ITNS, Italian Sardinian Wild Boar – ITWB2, Italian Wild Boar – ITWB1, Landrace population 1 – LDR1, Landrace population 2 – LDR2, NW European Wild Boar – NEWB, Pietrain population 1 – PIT1, Pietrain population 2 – PIT2, Polish Pulawska Spot – PLPS, Portuguese Bisaro – PTBI, South Balkan Wild Boar – SBWB, Spanish Chato Murciano – ESCM, Spanish Iberian – ESIB, **Turopolje – CROTS**, UK Berkshire – UKBK, UK British Saddleback – UKBS, UK Gloucester Old Spot – UKGO, UK Hampshire – UKHS, UK Large Black – UKLB, UK Tamworth – UKTA, USA Berkshire – USBK, USA Hampshire – USHS, and USA Poland China – USPC.

The distribution of the ROH inbreeding coefficients (F_ROH__>__4__Mb_) for all the analyzed breeds and wild populations is presented in Figure 3. The same figure also shows the contribution (%) of the “close” inbreeding (F_ROH__>__8__Mb_) caused by ancestors within seven generations relative to the total inbreeding level (F_ROH__>__4__Mb_). Among the considered pig breeds/populations, 65–89% of inbreeding came from ‘close’ inbreeding. “Close” inbreeding was significantly lower (*P* < 0.001) in wild boar individuals (mean F_ROH__>__8__Mb_ = 0.112; 95% confidence interval, CI = 0.094–0.128) than in domestic breeds (mean F_ROH__>__8__Mb_ = 0.172; 95% CI = 0.162–0.182). The difference remained significant (*P* < 0.001) even when two populations with high outlying inbreeding were excluded from the analysis (mean F_ROH__>__8__Mb_ = 0.156; 95% CI = 0.147–0.165). This was unexpected, since domestic pigs are bred based on pedigree information in order to avoid mating of relatives within six to seven generations. Extremely high inbreeding values (F_ROH__>__4__Mb_ = 0.400 and F_ROH__>__8__Mb_ = 0.332; Supplementary Figure S3) were observed in Turopolje pig, and such extreme inbreeding values were observed only in Hungarian Mangalitza (HUMA) (F_ROH__>__4__Mb_ = 0.415, and F_ROH__>__8__Mb_ = 0.371). An increased frequency of very long ROH (>30 Mb), showing increased close inbreeding, was also observed in Romanian and Hungarian Red Mangalitza pigs (Bâlteanu et al., 2019). In contrast, much lower inbreeding was observed in Black Slavonian pigs (F_ROH__>__4__Mb_ = 0.098 and F_ROH__>__8__Mb_ = 0.074).

### Analysis of Population Structure and Differentiation of Populations

The population differentiation was analyzed by pairwise F_ST_ values estimated across all populations from the final dataset of 32 breeds/populations (Supplementary Table S4). The mean F_ST_ estimate was 0.25, while all pairwise F_ST_ values from a selection of breeds are shown in Table 1. The F_ST_ values ranged from 0.07 (between the two Pietrain populations) to 0.40 (between Turopolje and Gloucester Old Spot pig breed). Genetic differentiation tends to be smaller between local pig breeds with a closer genetic history. The Black Slavonian breed showed a low mean F_ST_ value (0.21), consistent with its central position in the international dataset, while Turopolje pig had higher mean F_ST_ value (0.32), consistent with its peripheral position. The highest mean F_ST_ estimate among all breeds in this study was 0.33 for UK Tamworth; the lowest estimate was 0.18 for Czech Prestice. The observed values of genetic differentiation are comparable to the results of other studies.

**TABLE 1:** Genetic differentiation among pig breeds/populations based on F_ST_ estimates.

Breed/population	DEAS	ITCS	ITCT	ESIB	UKLB	LDR1	HUMA	NEWB	SBWB	CROWB	TRPR	USPC	UKBK	CROBS	F_ST_
Italy Cinta Senese – ITCS	0.25														0.29
Italy Casertana – ITCT	0.18	0.25													0.23
Spain Iberian – ESIB	0.21	0.22	0.19												0.23
UK Large Black – UKLB	0.21	0.30	0.23	0.25											0.26
Landrace – LDR1	0.19	0.30	0.23	0.28	0.27										0.27
Hungary Mangalica – HUMA	0.28	0.31	0.27	0.22	0.30	0.33									0.29
NW European Wild Boar – NEWB	0.26	0.28	0.25	0.19	0.29	0.31	0.28								0.26
South Balkan Wild Boar – SBWB	0.24	0.27	0.22	0.18	0.27	0.30	0.26	0.13							0.24
Croatia Wild Boar – CROWB	0.27	0.30	0.25	0.21	0.29	0.32	0.29	0.15	0.10						0.26
Czech Prestice – TRPR	0.08	0.21	0.15	0.17	0.17	0.18	0.23	0.22	0.20	0.22					0.18
USA Poland China – USPC	0.19	0.30	0.22	0.24	0.25	0.26	0.32	0.29	0.27	0.30	0.15				0.25
UK Berkshire – UKBK	0.25	0.35	0.27	0.29	0.29	0.30	0.34	0.33	0.31	0.34	0.21	0.28			0.29
Black Slavonian pig – CROBS	0.17	0.24	0.18	0.18	0.20	0.23	0.24	0.22	0.20	0.23	**0.13**	0.21	0.24		0.21
Turopolje pig – CROTS	0.30	0.35	0.29	0.26	0.33	0.34	0.34	0.32	0.30	0.33	**0.25**	0.35	0.37	0.28	0.32

*Pairwise F_ST_ estimates (Weir and Cockerham, 1984) presenting genetic differentiation among 14 selected breeds/populations and average F_ST_ estimates for each breed/population (right column).*

**FIGURE 4:**
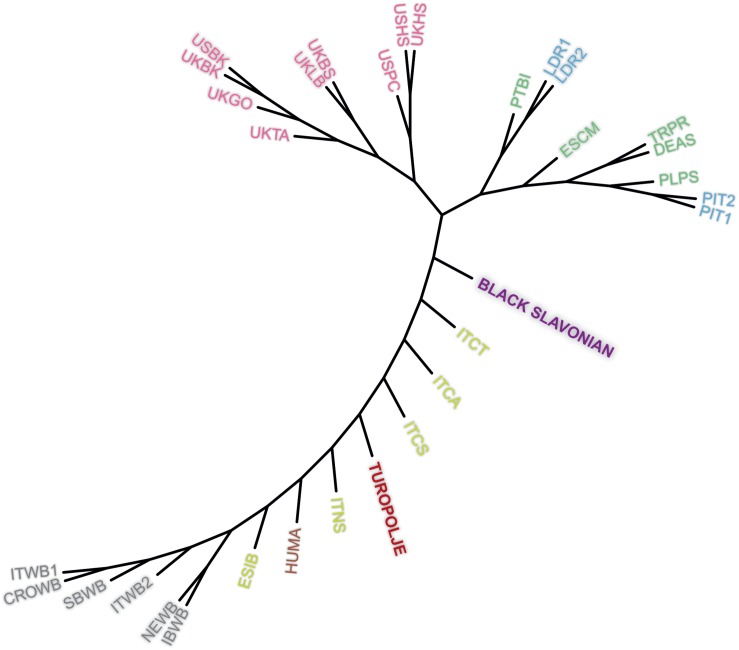
Neighbor-joining tree based on the Reynold’s genetic distances for selected 32 pig and wild boar populations. The breeds on the plot are: **Black Slavonian**, Croatian Wild Boar – CROWB, Czech Prestice – TRPR, German Angler Sattleschwein – DEAS, Hungarian Mangalitza – HUMA, Iberian Wild Boar – IBWB, Italian Calabrese – ITCA, Italian Casertana – ITCT, Italian Cinta Senese – ITCS, Italian Nera Siciliana – ITNS, Italian Sardinian Wild Boar – ITWB2, Italian Wild Boar – ITWB1, Landrace population 1 – LDR1, Landrace population 2 – LDR2, NW European Wild Boar – NEWB, Pietrain population 1 – PIT1, Pietrain population 2 – PIT2, Polish Pulawska Spot – PLPS, Portuguese Bisaro – PTBI, South Balkan Wild Boar – SBWB, Spanish Chato Murciano – ESCM, Spanish Iberian – ESIB, **Turopolje**, UK Berkshire – UKBK, UK British Saddleback – UKBS, UK Gloucester Old Spot – UKGO, UK Hampshire – UKHS, UK Large Black – UKLB, UK Tamworth – UKTA, USA Berkshire – USBK, USA Hampshire – USHS, and USA Poland China – USPC.

Genetic differentiation among 32 breeds/populations was further illustrated by unrooted neighbor-joining tree based on Reynolds genetic distances (Figure 4). The presented tree clearly shows differentiation of wild boar populations from commercial breeds while a number of indigenous breeds are presented between this separation of two extreme groups (wild boar populations versus commercial breeds) in a succeeding manner, starting with Iberian pig (ESIB) as the closest breed to the wild populations and ending with Black Slavonian breed as the closest breed to the commercial breeds. In this separation route Turopolje pig is positioned in the middle.

### Identification of Adaptive Signatures of Selection

In order to identify loci specific to Croatian indigenous breeds and therefore more useful for conservation efforts, we separately calculated the locus-wise F_ST_ values between Croatian local breeds and modern pig breeds (Landrace and Pietrain). We selected 30 genome-wide SNPs with the highest F_ST_ values for Black Slavonian and Turopolje pigs (Supplementary Figures S1, S2). Genes located within the genomic regions of SNPs with extremely high F_ST_ were identified as candidate genes that could help in future conservation programs. Most likely polymorphisms in these genes are the consequence of breed adaptation to environmental and human demands. For Black Slavonian pig, we identified important genes associated with steroid receptor activity, such as CYP-40 on porcine chromosome SSC8 (Ratajczak et al., 2015); meat-to-fat ratio in pigs, DEAF1 on SSC2 (Falker-Gieske et al., 2019); growth traits in cattle, KSR2 on SSC14 (Puig-Oliveras et al., 2014); animal organ and system development in pigs, SEZ6L on SSC14 (Kwon et al., 2019); hematological parameters in pigs, RHOBTB1 on SSC 14 (Bovo et al., 2019); female reproduction in mice, CDK1 on SSC 14 (Adhikari et al., 2016); salivary secretion in pigs, KCNMA1 on SSC14 (Li et al., 2013); milk fat percentage in buffaloes, KCTD8 on SSC8 (de Camargo et al., 2015); back fat thickness in pigs, RIMS4 on SSC17 (Lee and Shin, 2018); carcass length in pigs, SPTLC2 on SSC7 (Falker-Gieske et al., 2019); muscle fiber types in pigs, MYO18B on SSC14 (Ropka-Molik et al., 2018); and fatty acid profiles in cattle, RAPGEF2 on SSC8 (Cesar et al., 2014).

For Turopolje pig, we identified candidate genes associated with fatty acid metabolism in pigs, such as PEX11A on SSC7 (Huang et al., 2017); carcass traits in cattle, WDR93 on SSC7 (Silva et al., 2017); number of ribs in pigs, MESP1 on SSC7 (Zhu et al., 2015); meat-to-fat ratio in pigs, DEAF1 on SSC2 (Falker-Gieske et al., 2019); pregnancy rate in pigs, PPID on SSC8 (Gu et al., 2014); steroid receptor activity, CYP-40 on SSC8 (Ratajczak et al., 2015); brain development in horses, DLGAP1 on SSC6 (Schubert et al., 2014); salivary secretion in pigs, KCNMA1 on SSC14 (Li et al., 2013); reproduction in pigs, CWH43 on SSC8 (He et al., 2017); bone weight in cattle, FAM184B on SSC8 (Xia et al., 2017) and cardiovascular disease (Pérez-Montarelo et al., 2014); spermiogenesis in mouse, AMPH on SSC9; boar taint, NWD2 on SSC8 (Drag et al., 2018); melanocyte function in dogs, ARHGAP12 on SSC10 (Kluth and Distl, 2013); female pregnancy in pigs, RAPGEF2 on SSC8 (Pérez-Enciso et al., 2009); growth traits in cattle, KSR2 on SSC14 (Puig-Oliveras et al., 2014); vascular smooth muscle contraction in sheep, SPSB4 on SSC13 (Yang et al., 2016); and back-fat fatty acid composition, APBB1IP on SSC10 (Zappaterra et al., 2018).

In addition, we identified several SNPs in the two Croatian breeds that were located in non-coding intergenic regions and that were present in various pig breeds as well as to other domestic animal species, including cattle, sheep and horse. In Black Slavonian pig, the following 12 SNPs were identified: ASGA0012664, ALGA0082391, SIRI0000509, ALGA0115258, ALGA0008072, ASGA0038761, ASGA0080338, ALGA0098790, ASGA0039781, ASGA0039779, M1GA0015147, and MARC0003342. In Turopolje pig, the following nine SNPs were identified: MARC0067231, ALGA0115258, M1GA0015147, ASGA0038761, ALGA0048121, MARC0085941, ASGA0042725, ASGA0038765, and SIRI0000509.

To provide additional support to the identification of genome regions with adaptive selection signatures, we also performed several tests that are used in the identification of selection signatures such as eROHi, iHS and Rsb analysis. The overall results of the selection signature analyses are presented in Supplementary Table S5. Among all approaches performed, F_ST_ and Rsb analyses are the most similar by the concept as they are both looking for genome segments that are selected in indigenous breeds in contrast to commercial populations, while eROHi and iHS analyses are based on the identification of adaptive selection signatures from genomic information of the single population.

We have not identified any significant SNP overlapping between F_ST_ and Rsb analyses neither in Black Slavonian nor in Turopolje pig population. However, when we were looking for the overlapping results between F_ST_ and eROHi analysis, three significant SNPs (MARC0058238, MARC0003342, and ALGA0077279, all on SSC14) pointing to the adaptive selection signals, were observed in Black Slavonian breed while only one such significant SNP (ALGA0036219 on SSC6) was observed in Turopolje breed. The first SNP for Black Slavonian was previously described (MARC0058238, located in the MYO18B genomic region on SSC14, which is found to be associated with muscle fiber types in pigs- Ropka-Molik et al., 2018). Second (MARC0003342) and third (ALGA0077279) SNP identified in Black Slavonian pig are located in non-coding intergenic region present in various domestic animal species, together with the SNP (ALGA0036219 on SSC6) found in Turopolje pig. We identified one additional SNP (ASGA0060892 on SSC14) with significant selection signal obtained in both eROHi and iHS analyses in Turopolje pig. This variant, located in PEBP4 gene region, is associated with hematological traits in pigs (Bovo et al., 2019) and has been shown to differentiate Chinese local breeds from Large White pigs (Li et al., 2014).

## Discussion

Over the last hundred years, strong demand for animal protein and economic efficiency, combined with globalization and market competition, have intensified pig breeding and selection, leading to the domination of several commercial breeds such as Large White, Duroc, Landrace, Hampshire, and Pietrain. In the last few decades, many valuable local breeds have gone extinct or are on the brink of extinction. Conserving these species is important for maintaining genetic diversity to promote long-term selection progress (Bruford et al., 2015).

Black Slavonian and Turopolje pigs are Croatian local indigenous breeds that are well adapted to harsh environments and should be preserved from extinction as they can contribute to the overall adaptive genetic potential. In this study, based on high-throughput genomic information, Black Slavonian and Turopolje pig breeds were genetically compared with many internationally relevant breeds, as well as with several wild boar populations. MDS multivariate analysis and unsupervised clustering showed that both breeds have complex but close genetic relatedness with other European pig breeds, and can be considered part of the living European livestock (pig) heritage (Figures 1 and 2). The Black Slavonian pig appears to be more influenced by the classical West European breeds, while the Turopolje pig clusters with the Mediterranean pig breeds in vicinity to the cluster representing wild boars (Figures 1, 2). Still, the algorithm implemented in our STRUCTURE analysis was able to make a distinction among Turopolje pig (at *K* = 13), Black Slavonian pig (at *K* = 18), and other European pig breeds. Turopolje pig showed a low level of admixture with commercial pigs, while Black Slavonian pig showed greater and more variable admixture (Figure 2). The admixture contributions in the Black Slavonian pig originated from several equally contributing clusters belonging to different commercial breeds. We speculate that these are signals of admixture with some of the modern pure breed or hybrid pigs commercially reared in Slavonia, pointing to the need for further maintenance of systematic breeding programs for breed consolidation and recovery. High inbreeding values, particularly the recent ones, were obtained for the Turopolje pig. With the exception of the Hungarian Mangalitza breed, such high inbreeding values have not been reported for the other breeds analyzed here (Saura et al., 2013; Schäler et al., 2020). The observed values exceed considerably even the expected inbreeding that would result from full sib or parent-offspring mating, and they seriously threaten the survival of the breed. A much better situation, with a relatively low inbreeding level, was observed in Black Slavonian pig, even if the breed went through a severe bottleneck in the 1990’s. The presence of admixture signals could certainly have an impact on the observed inbreeding level, but only for the admixed individuals. Thus, we think that the observed inbreeding level is the consequence of the recent breeding program and pedigree-controlled mating strategy performed in the last decade, according to which only sows and boars with known ancestry and acceptable coefficient of relationship were allowed to mate.

A recent study analyzed genomic diversity, linkage disequilibrium and selection signatures in European local pig breeds, including Black Slavonian and Turopolje pig (Muñoz et al., 2019). Their aims were slightly different from those of the present study, and their analyses were oriented toward European local breeds more generally. In contrast, we were interested in conservation genomics and estimation of admixture and genomic inbreeding in the two indigenous Croatian breeds. Thus, their analysis relied on GeneSeek^®^ GGP Porcine HD Genomic Profiler v1 markers, while ours relied on PorcineSNP60 v2 markers. We were aware that the sample size in our study was small for the reliable estimation of gametic (Ne_GD_) or/and linkage disequilibrium (Ne_LD_) effective population size. Their analysis estimated very small effective population size based on linkage disequilibrium (N_eLD_) in Black Slavonian pigs (N_eLD_ = 33) and Turopolje pigs (N_eLD_ = 10) for the current generation, although the estimates of the contemporary N_eLD_ population size are quite sensitive (Corbin et al., 2012). Future work, on a larger sample size should estimate these parameters because they are important for conservation assessment of these Croatian indigenous breeds.

Nevertheless, the sample sizes of breeds or populations in the present study are comparable to those in similar studies (Burgos-Paz et al., 2013; Goedbloed et al., 2013; Decker et al., 2014) and were appropriate for analyses on MDS, population structure and admixture with the STRUCTURE algorithm, estimation of genomic inbreeding and identification of breed-specific genome regions. A larger sample size would narrow the CI of the estimated inbreeding level but not alter our conclusions, since the estimated marginal values of the confidence intervals in Turopolje pig were extremely high (the 95% CI was 0.342–0.459 for F_ROH__>__4__Mb_, and the 95% CI was 0.280–0.383 for F_ROH__>__8__Mb_), whereas those obtained in Black Slavonian pig were relatively low (the 95% CI was 0.071–0.123 for F_ROH__>__4__Mb_, and the 95% CI was 0.050–0.097 for F_ROH__>__8__Mb_) compared to the estimated inbreeding level in other breeds in the present study as well as in other studies (Saura et al., 2013; Schäler et al., 2020).

In addition, it is important to highlight that Turopolje pig population consists of 124 sows and 17 boars (Croatian Agricultural Agency, 2017). Also, the ascertainment bias could have influenced the analyses performed, since both Black Slavonian and Turopolje pigs are local breeds that were not included in the development of the Illumina PorcineSNP60 v2 Genotyping BeadChip. However, such influence is likely to be minimal while our findings should be verified in studies based on whole-genome sequencing.

We obtained additional insights into the genetic background of Croatian local pigs through the identification of genomic regions that show a high level of differentiation (extreme F_ST_) between the Croatian indigenous pigs and commercial modern animals. Genes identified within those regions are likely to have important adaptive functions and therefore are suitable for traceability studies to protect and promote products derived from Black Slavonian and Turopolje pig. In addition to their expected functions, these candidate genes have been associated with production or carcass traits in Black Slavonian pig (DEAF1, KSR2, RIMS4, and SPTLC2) and Turopolje pig (WDR93, MESP1, DEAF1, and KSR2), as well as with reproduction and system development in Black Slavonian pig (SEZ6L, RHOBTB1, CDK1, and KCNMA1) and Turopolje pig (PPID, KCNMA1, CWH43, RAPGEF2, SPSB4, and APBB1IP). In identifying adaptive selection signals with F_ST_ analysis we were extremely conservative as only 30 SNPs with highest F_ST_ values were considered significant. We wanted to minimize the number of false positive selection signatures. Thus, we have performed additional analyses toward identification of selection signals (eROHi, iHS, and Rsb). The presence of selection signatures obtained by F_ST_ analysis was confirmed for three genome regions in Black Savonian breed and one genome region in Turopolje breed. Additional selection signature has been identified in PEBP4 gene region (placed on SSC14) in Turopolje breed as significant signals were obtained by eROHi and iHS analyses. Assuming that the necessary data become available, future work may wish to take a more comprehensive approach, at least for the relatively large Black Slavonian population, by estimating breeding values for traits of conservation interest and combining those estimates with F_ST_ values to detect conservation-relevant SNPs (Zhang et al., 2014).

## Conclusion

In conclusion, our results show that Black Slavonian and Turopolje pigs are distinct breeds genetically related to other European pig breeds. Uncontrolled breeding is likely to reduce the genomic diversity of European pig breeding capacity and threaten the cultural heritage of these breeds. Although conservation planning has already been implemented for the Black Slavonian pig, and our results suggest that such planning has benefited the breed, future actions toward admixture consolidation and management are required. The conservation status of the Turopolje pig is alarming and an urgent conservation plan is needed. The two local breeds in this study currently make only a marginal contribution to commercial pig production, yet we need to protect the genetic variability of these local breeds to guarantee necessary genetic diversity for the future. The identification of breed specific genome regions with extreme F_ST_ values will enable protection and promotion of commercial products derived from Black Slavonian and Turopolje pigs.

## Data Availability Statement

This manuscript contains previously unpublished data available at Dryad (https://doi.org/10.5061/dryad.wpzgmsbhq).

## Ethics Statement

All procedures used for this study involving animals were in accordance with the guidelines for animal welfare defined by the Croatian Ministry of Agriculture.

## Author Contributions

VC-C and IC conceived the research. MČ, VO, and LI conducted the field work and laboratory analyses. BL, MF, and DŠ analyzed the data. BL, VC-C, and IC wrote the manuscript. All authors drafted and approved the current version of the manuscript.

## Conflict of Interest

The authors declare that the research was conducted in the absence of any commercial or financial relationships that could be construed as a potential conflict of interest.

## Footnotes

1 http://www.ensembl.org/Sus_scrofa, Sscrofa 10.2 assembly
