# Supplementary material for: Conservation Genomic Analysis of the Croatian Indigenous Black Slavonian and Turopolje Pig Breeds
Source: Front Genet. 2020 Mar 31;11:261. doi: 10.3389/fgene.2020.00261 (PMC7136467; doi:10.3389/fgene.2020.00261)
Supplement: Supplementary file 6 [file Data_Sheet_1.PDF]

Supplementary Figure S1.

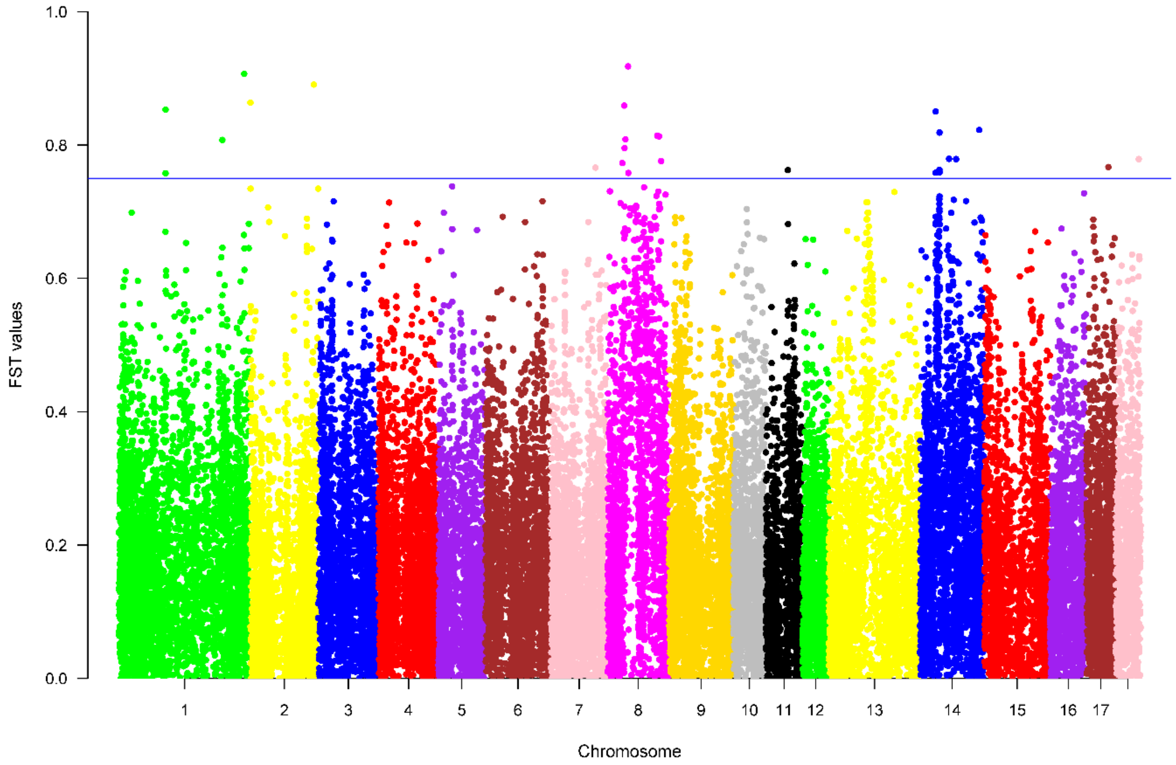

Genome-wide analysis of global F_{ST} between Black Slavonian pig and modern pig breeds (Landrace and Pietrain). Manhattan plot of genome-wide F_{ST} values between the Black Slavonian pigs and modern pig breeds. F_{ST} values are shown on the y axis, and genomic locations on the x axis. The different chromosomes are shown in color range.

Supplementary Figure S2.

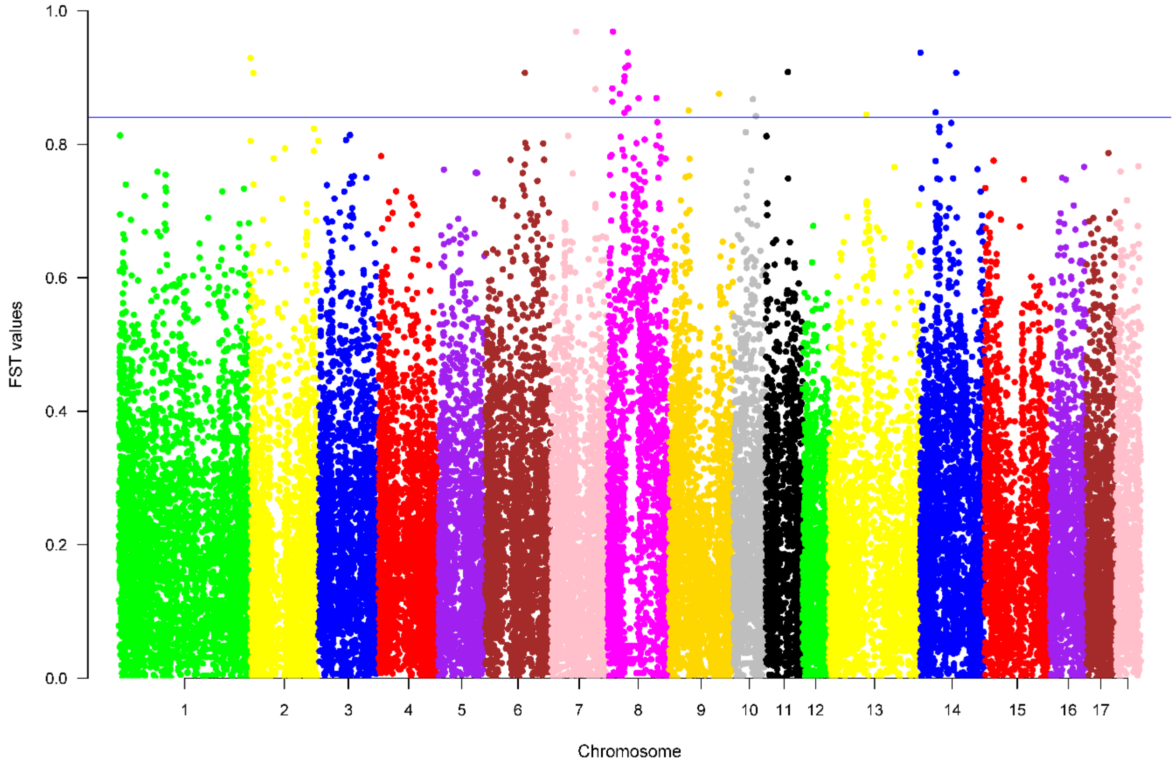

Genome-wide analysis of global F_{ST} between Turopolje pig and modern pig breeds (Landrace and Pietrain). Manhattan plot of genome-wide F_{ST} values between Turopolje pig and modern pig breeds. F_{ST} values are shown on the y axis, and genomic locations on the x axis. The different chromosomes are shown in color range.

Supplementary Figure S3.

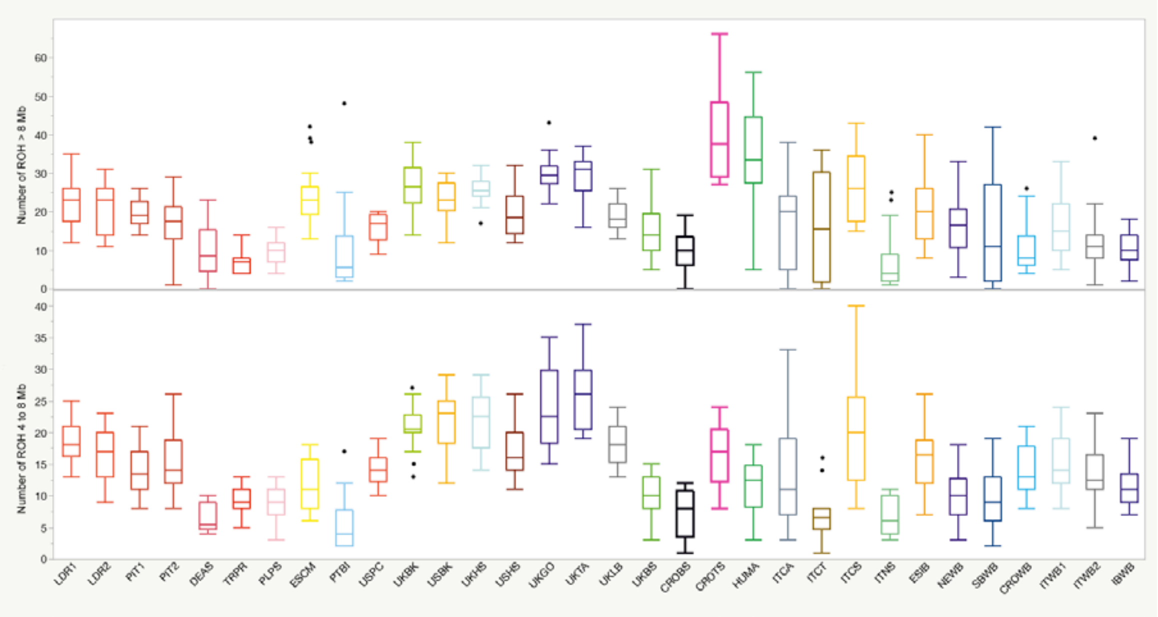

The distribution of the ROH inbreeding coefficients (F_{ROH} 4 to 8 Mb; F_{ROH} >8 Mb) for the analyzed pig populations.

Supplementary Figure S4**.**

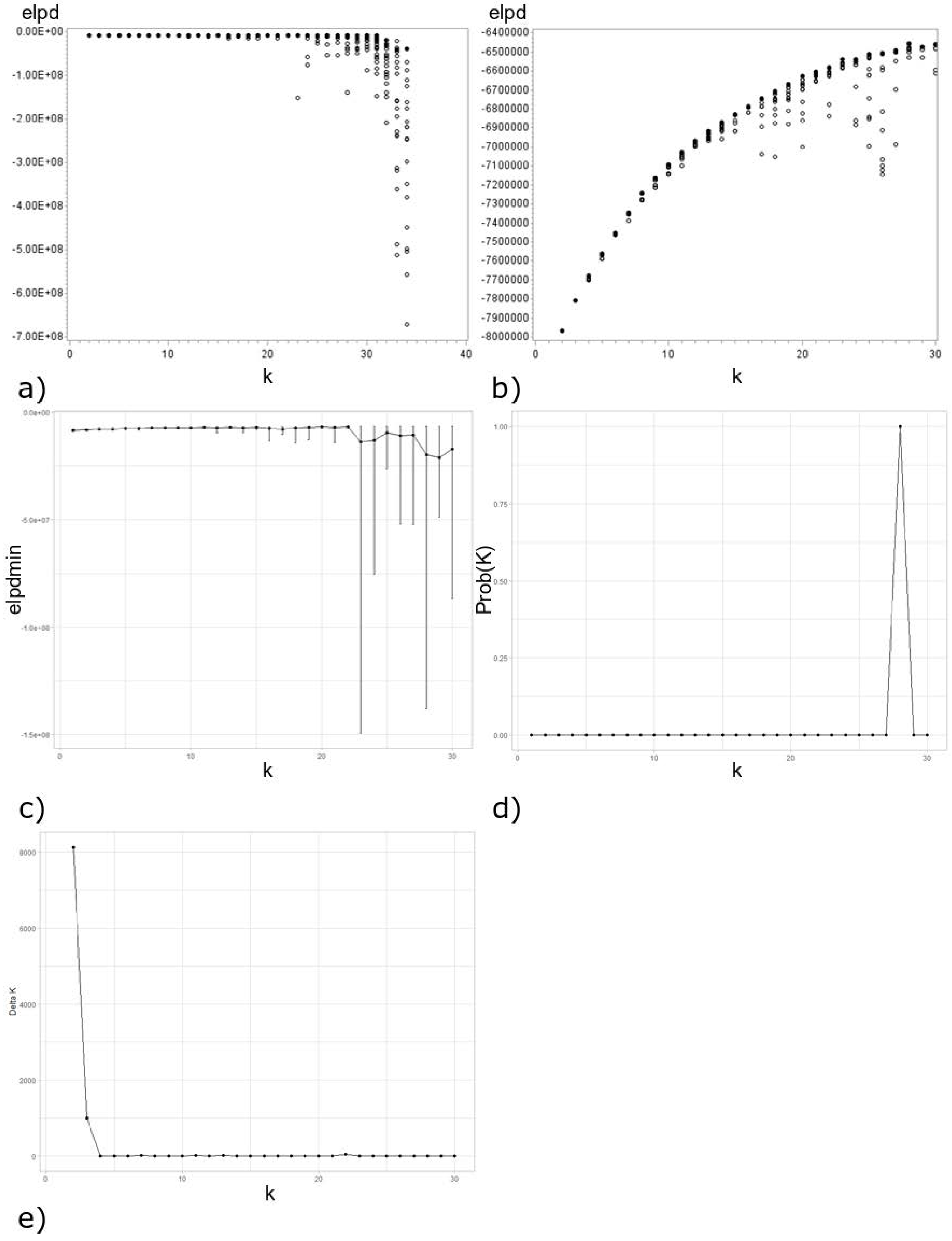

Figure S4 Plots representing the results of the 20 runs per model in the structure population analysis used to determine the most likely number of clusters (K) of the analysed pig populations.

a) K plot showing the estimated ln probability of data (elpd) for each k (k=1 to 34) and each run.
b) K plot showing the estimated ln probability of data for each k used in the further analysis (k=2 to 30) and selected runs where the smallest elpd difference from the other elpd values included in the plot, for the specific k, is less than -200 000 where k=16 to 30, and less than -100 000 where k=1 to 15.
c) Mean likelihood (±SD) over the 20 runs for each k value tested (k=1 to 30), obtained by pophelper (Francis, 2017).
d) Probability by K plot using median values of Ln(Pr Data) the k for which Pr(K=k) is highest over the 20 runs for each k value tested (k=1 to 30), obtained by CLUMPAK (Kopelman et al., 2015).
e) Delta K curve (Delta K = mean(|L''(K)|) / stdev[L(K)]) estimated according to Evanno et al. (2005), over the 20 runs for each k value tested (k=1 to 30), obtained by CLUMPAK (Kopelman et al., 2015).

Other K plots

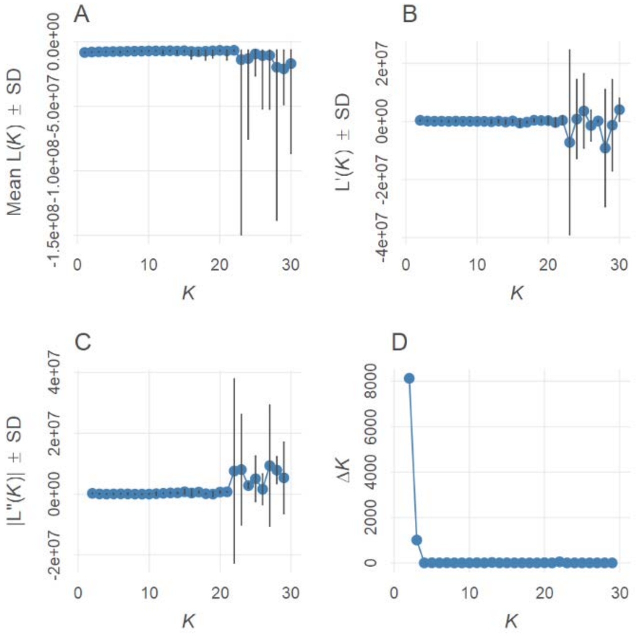

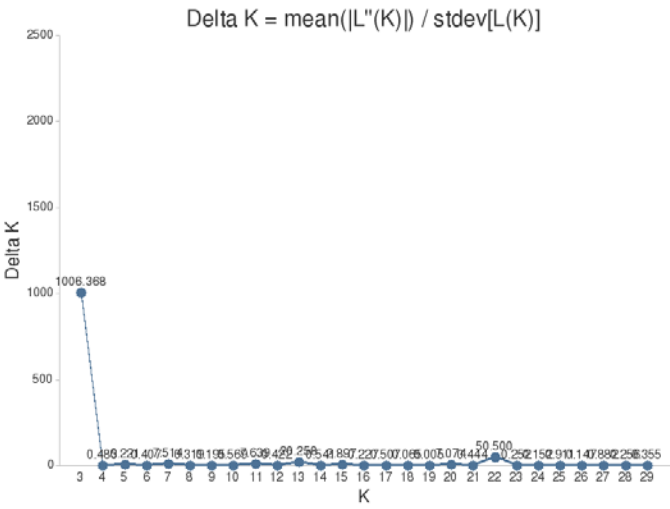

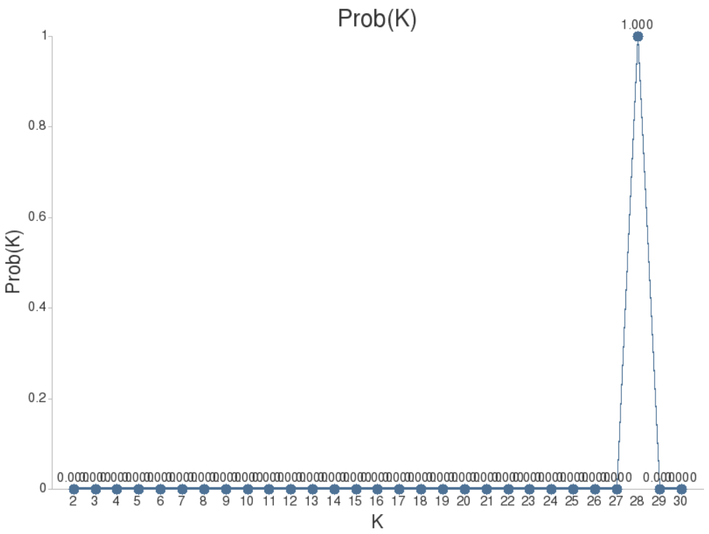
Prob(K)
1.000
Prob(K)
K
1
0.8
0.6
0.4
0.2
0
2 3 4 5 6 7 8 9 10 11 12 13 14 15 16 17 18 19 20 21 22 23 24 25 26 27 28 29 30

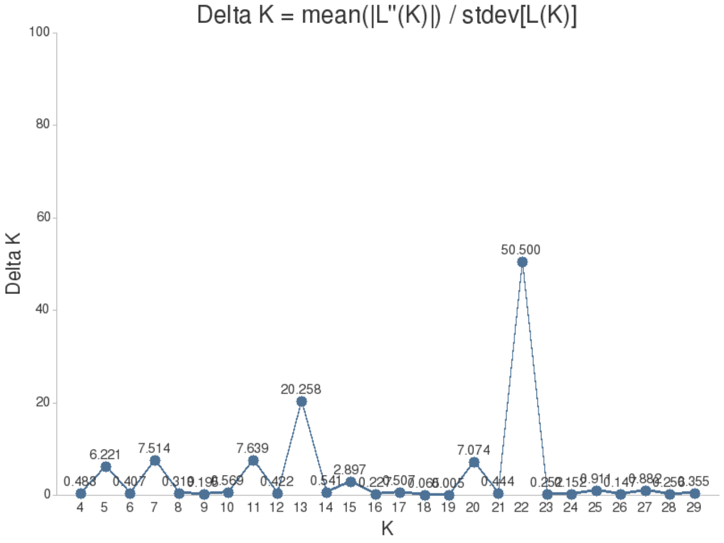
Delta K = mean(|L''(K)|) / stdev[L(K)]
Delta K
K
100
80
60
40
20
0
50.500
20.258
6.221
7.514
7.639
7.074
2.897
4 5 6 7 8 9 10 11 12 13 14 15 16 17 18 19 20 21 22 23 24 25 26 27 28 29

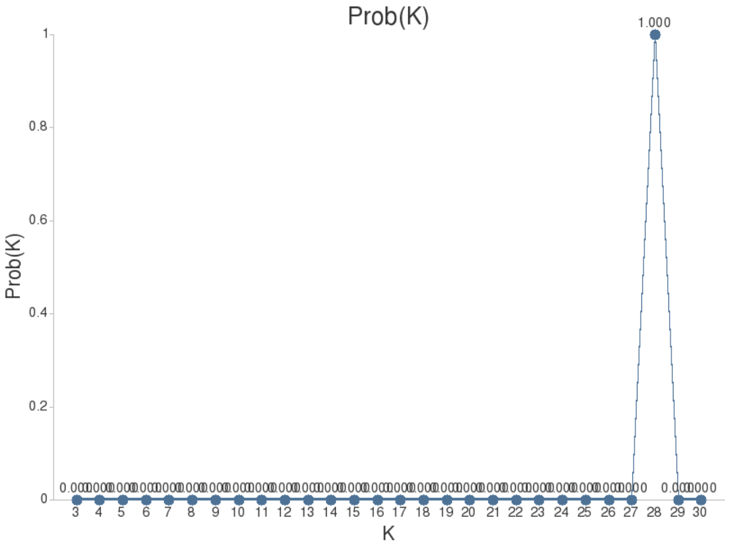
Prob(K)
1.000
Prob(K)
K
1
0.8
0.6
0.4
0.2
0
3 4 5 6 7 8 9 10 11 12 13 14 15 16 17 18 19 20 21 22 23 24 25 26 27 28 29 30

Structure analysis parameters

Analyses of the ln Pr(G|K) for the 20 runs performed for each K-value (1 to 34) showed high convergence for K values up to K = 24 and increasing variability, with decreasing values of ln Pr(G|K) for higher K values. The analyses of the populations over 20 runs per each of the 34 models showed a high range of ln Pr(G|K) and decreasing values from K = 34 (Supplementary Figure S4a). A closer inspection of the K plot showed that the estimated ln Pr(G|K) for each K used for further analysis (K = 1 to 30) increased if the runs were selected in the difference between the smallest and other ln Pr(G|K) values included in the plot. Values of ln Pr(G|K) were lower than -200,000 for K = 16 to 30, and lower than -100,000 for K = 1 to 15 (Supplementary Figure S4b). Furthermore, mean likelihoods (±standard deviation, SD) over the 20 runs for each K value showed the biggest decrease when K = 23, with a similar pattern for consecutive K values (Supplementary Figure S4c). The most appropriate number of clusters for 32 populations according to ΔK (8,125) was two (Supplementary Figure S4e). The secondary peak of ΔK (1,006) was obtained for K = 3. Consecutive ΔK values with lower but observable magnitudes were K = 22 (50.5) and K = 13 (20.58). The K graph (see Supplementary Figure S4d) revealed that the model with K = 28 was the best, with Pr(K) = 1.

Figure 2 shows the results of models with K = 3, 13, 18, or 28 to determine the population structure and admixture of Black Slavonian and Turopolje pigs with respect to other domestic breeds and wild boar populations. All 20 runs of models K = 3 and K = 18 were consistent and grouped together, with respective average ln Pr(G|K) values of -7,808,713 and -7,305,082. The runs of models after K = 4 showed different clustering outcomes, with an additional 1-3 minor modes. The model K = 13 showed a major mode of 10 runs, with an average ln Pr(G|K) equal to -6,939,055, and two minor modes. The first minor mode consisted of seven runs with average ln Pr(G|K) equal to -6,928,876, and the second minor mode consisted of three runs with average ln Pr(G|K) equal to -6,921,163. Model K = 28 showed a major mode consisting of 11 runs with the average ln Pr(G|K) equal to -17,649,905, and two minor modes. The first minor mode consisted of four runs with average ln Pr(G|K) equal to -40,880,514, and the second minor mode consisted of five runs with average ln Pr(G|K) equal to -7,347,507.
